# Association between Serum Glycated Hemoglobin Levels and Female Infertility: A Cross-Sectional Survey and Genetic Approach

**DOI:** 10.3390/ijms25179668

**Published:** 2024-09-06

**Authors:** Chung-Chih Liao, Chun-I Lee, Ke-Ru Liao, Jung-Miao Li

**Affiliations:** 1 Department of Post-Baccalaureate Veterinary Medicine, College of Medical and Health Science, Asia University, Taichung 41354, Taiwan; ccliao@asia.edu.tw; 2 Chuyuan Chinese Medicine Clinic, Taichung 40455, Taiwan; 3 Institute of Medicine, Chung Shan Medical University, Taichung 40201, Taiwan; adoctor0402@gmail.com; 4 Division of Infertility, Lee Women’s Hospital, Taichung 40652, Taiwan; 5 Department of Obstetrics and Gynecology, Chung Shan Medical University Hospital, Taichung 40201, Taiwan; 6 Department of Neurology, Yuanlin Christian Hospital, Changhua 51052, Taiwan; u9401407@gmail.com; 7 School of Chinese Medicine, College of Chinese Medicine, China Medical University, Taichung 40402, Taiwan; 8 Department of Chinese Medicine, China Medical University Hospital, Taichung 40447, Taiwan

**Keywords:** female infertility, glycated hemoglobin, Mendelian randomization, cross-sectional study, reproductive health

## Abstract

Female infertility affects a significant portion of the population, and recent studies suggest a potential link between glycemic control and reproductive health. This study investigates the association between serum glycated hemoglobin (HbA1c) levels and female infertility, utilizing data from the NHANES 2017–2020 and Mendelian randomization (MR) analysis. A cross-sectional study was conducted with 1578 women aged 20–45 who attempted pregnancy for at least one year. Serum HbA1c levels were analyzed in relation to infertility status, with multivariable logistic regression models adjusting for covariates such as age, body mass index, race/ethnicity, education, marital status, hypertension, and hyperlipidemia. Higher HbA1c levels were significantly associated with increased infertility risk. Each 1% increase in HbA1c was linked to higher odds of infertility (adjusted OR: 1.40, 95% CI: 1.15–1.69, *p* = 0.003). HbA1c levels ≥ 6.5% showed the strongest association. MR analysis employed single-nucleotide polymorphisms as instrumental variables to assess the causal relationship between HbA1c and infertility, confirming a causal relationship between higher genetically predicted HbA1c levels and infertility (OR: 1.82, 95% CI: 1.33–2.49, *p* = 0.00018). Sensitivity analyses supported the robustness of these findings. Elevated HbA1c levels are associated with an increased risk of female infertility, suggesting the importance of glycemic control in reproductive health management.

## 1. Introduction

Infertility is a pervasive public health issue, impacting millions of women globally and posing significant physical, emotional, and financial burdens. Defined as the inability to conceive after one year of regular, unprotected intercourse, infertility’s multifactorial nature encompasses genetic, environmental, and lifestyle components [1,2]. Recently, metabolic factors such as glycemic control have emerged as potential contributors to reproductive health outcomes [3]. Serum glycated hemoglobin (HbA1c), a reliable indicator of long-term glucose regulation, has been extensively studied in relation to diabetes and cardiovascular diseases [4]. However, its potential impact on female infertility has not been thoroughly investigated, warranting further exploration.

The biological plausibility of a link between HbA1c levels and female infertility stems from the detrimental effects of hyperglycemia on various physiological processes. Chronic hyperglycemia can induce oxidative stress and inflammatory responses, which in turn may impair ovarian function, disrupt endometrial receptivity, and hinder embryo development [5,6]. Evidence suggests that women with metabolic disorders such as diabetes and polycystic ovary syndrome (PCOS)—both characterized by insulin resistance and elevated HbA1c levels—exhibit a higher prevalence of infertility [6]. Despite these associations, direct investigations into HbA1c levels and infertility in the general female population remain sparse.

The National Health and Nutrition Examination Survey (NHANES) 2017–2020, conducted by the CDC, offers comprehensive health and nutritional data, including detailed reproductive health information and biochemical measures. This dataset provides a unique opportunity to explore the association between HbA1c levels and infertility in women of reproductive age. Additionally, we employ Mendelian randomization (MR) analysis, using genetic variants as instrumental variables (IVs), to mitigate confounding factors and reverse causation. This research leverages NHANES data and MR analysis to investigate the potential causal relationship between serum glycated hemoglobin levels and female infertility.

## 2. Results

### 2.1. Basic Characteristics of Study Participants

A total of 1578 female participants aged 20–45 years were included in this study, comprising 192 infertility cases and 1386 normal controls. The baseline characteristics of the study population are presented in Table 1. There were statistically significant differences between the two groups in terms of age, marital status, BMI, hyperlipidemia, and HbA1c levels (all *p* < 0.05). Specifically, the infertility group was older (33.46 ± 6.35 vs. 32.22 ± 7.65 years, *p* = 0.036), had a higher BMI (32.76 ± 9.04 vs. 29.36 ± 8.48 kg/m^2^, *p* < 0.001), and a higher prevalence of hyperlipidemia (20.19% vs. 13.46%, *p* = 0.015). Additionally, a higher percentage of the infertility group was married or living with a partner (79.14% vs. 56.83%, *p* < 0.001). The HbA1c levels in the infertility group (5.57 ± 0.99%) were significantly higher than in the normal group (5.32 ± 0.59%) (*p* < 0.001). There were no significant differences in race/Hispanic origin, education level, or hypertension between the two groups.

### 2.2. HbA1c Levels and Infertility

Table 2 presents the associations between HbA1c levels and infertility across three different models. For HbA1c as a continuous variable, each 1% increase in HbA1c was significantly associated with a higher risk of infertility. In Model 1, the odds ratio (OR) was 1.48 (95% confidence interval [CI] 1.21–1.81, *p* < 0.001). This association persisted in Model 2 (OR 1.57, 95% CI 1.25–1.98, *p* < 0.001) and Model 3 (OR 1.40, 95% CI 1.15–1.69, *p* = 0.003), even after adjusting for various covariates. When HbA1c was categorized, using HbA1c < 5.7% as the reference, Model 1 showed that HbA1c levels between 5.7% and 6.4% had an OR of 1.73 (95% CI 1.09–2.75, *p* = 0.029), and levels ≥ 6.5% had an OR of 3.62 (95% CI 1.82–7.20, *p* = 0.001). In Model 2, the ORs were 1.78 (95% CI 1.03–3.07, *p* = 0.040) for HbA1c 5.7%–6.4% and 4.43 (95% CI 2.03–9.67, *p* = 0.001) for HbA1c ≥ 6.5%. In Model 3, the ORs were 1.34 (95% CI 0.76–2.36, *p* = 0.261) for HbA1c 5.7%–6.4% and 3.32 (95% CI 1.50–7.36, *p* = 0.007) for HbA1c ≥ 6.5%. Overall, higher HbA1c levels were significantly associated with an increased risk of infertility, both as continuous and categorical variables, with the strongest associations observed at HbA1c levels ≥ 6.5%.

### 2.3. The Causal Relationship of HbA1c and Female Infertility

Given the significant association observed between HbA1c levels and female infertility in the multivariable regression models, we further analyzed the potential causal relationship between these two factors. After harmonizing the SNP effects, we identified 17 and 23 SNPs as IVs for HbA1c and female infertility, respectively (Supplementary Tables S1 and S2). Each F-statistic was greater than 10, indicating that our IVs were not subject to weak instrument bias. Cochran’s Q test and the MR-Egger intercept suggested no evidence of pleiotropy or horizontal heterogeneity in the bidirectional MR analysis (Table 3). Additionally, MR-PRESSO analysis detected no horizontal pleiotropy effects or outliers affecting the relationship between HbA1c levels and female infertility.

**Figure 1:**
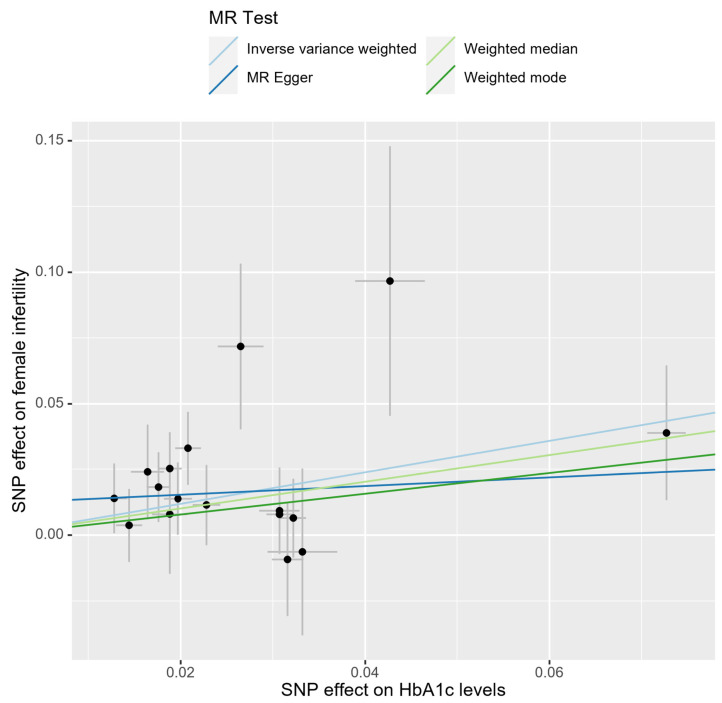
Scatter plot of causal estimates for HbA1c levels on female infertility.

**Figure 2:**
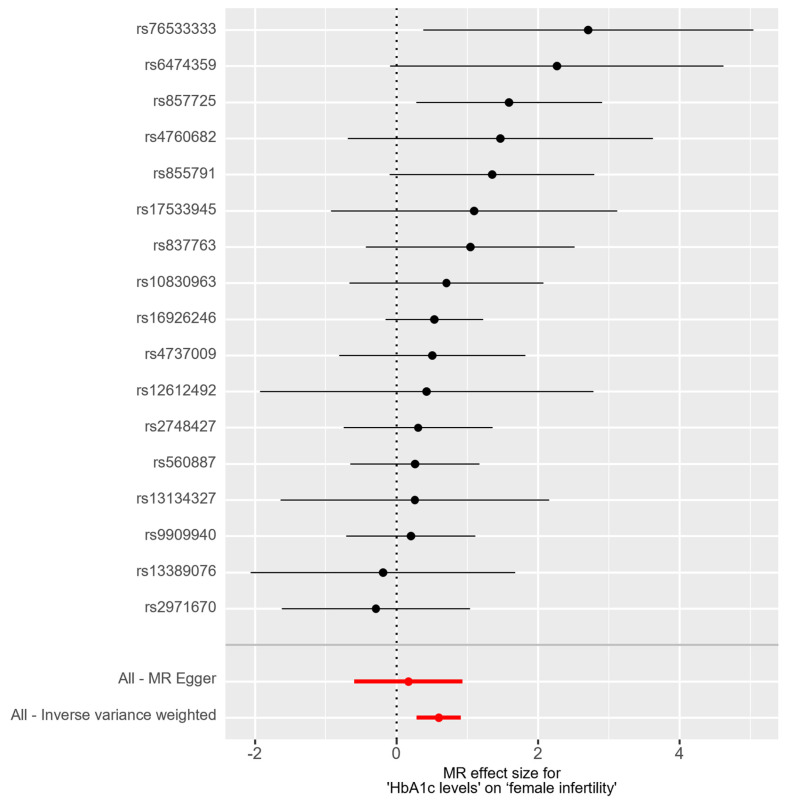
Forest plot of causal estimates for HbA1c levels on female infertility.

**Figure 3:**
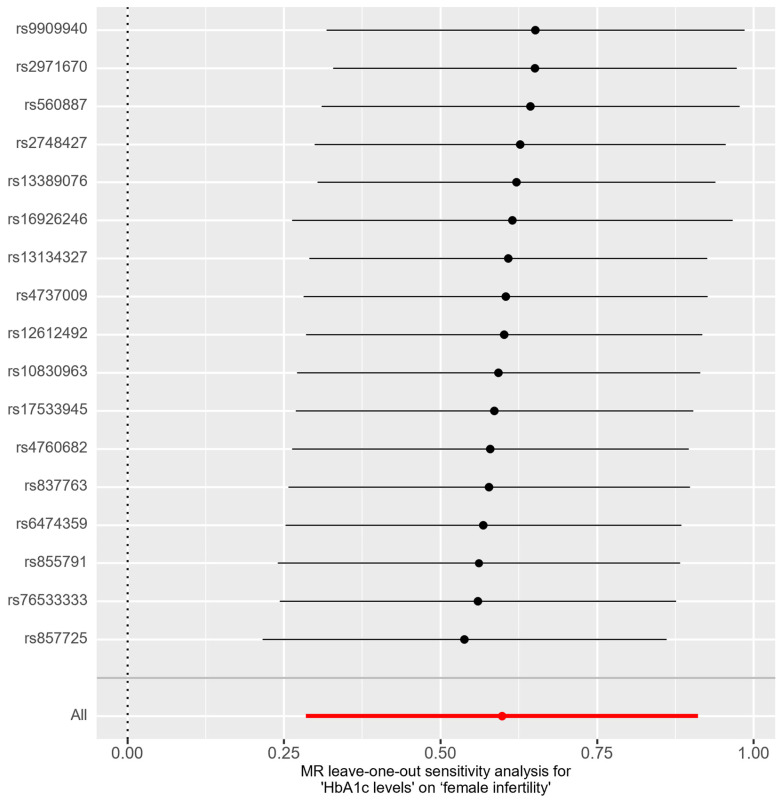
Leave-one-out analysis for association between HbA1c levels and female infertility.

In the bidirectional MR analysis, we applied several alternative MR methods to assess the causal relationships. Specifically, the primary analysis method, Inverse-Variance Weighted (IVW), yielded a significant causal association between genetically predicted HbA1c levels and female infertility [odds ratio (OR): 1.82, 95% confidence interval (CI): 1.33–2.49, *p* = 0.00018]. The results were consistent in terms of the direction and magnitude of the causal effect across various MR methods. Comprehensive scatter and forest plots for these analyses are shown in Figure 1 and Figure 2, respectively. The leave-one-out analysis indicated that no single SNP was responsible for driving the observed associations (Figure 3). In the reverse MR analysis, assessing the effect of female infertility on HbA1c levels, we found no genetic evidence of a causal effect (Supplementary Table S3).

## 3. Discussion

This study aimed to explore the association and potential causal relationship between HbA1c levels and female infertility using data from the NHANES 2017–2020 survey and a bidirectional MR analysis. Our results indicate that elevated HbA1c levels are significantly associated with an increased risk of female infertility, and the MR analysis supports a causal relationship between higher HbA1c levels and infertility.

The cross-sectional analysis revealed that women with higher HbA1c levels were more likely to report infertility. This finding persisted even after adjusting for various covariates, including age, race/ethnicity, education level, marital status, BMI, hypertension, and hyperlipidemia. The strongest associations were observed at HbA1c levels of 6.5% or higher, suggesting that hyperglycemia may play a critical role in reproductive health. Our MR analysis, which uses genetic variants as IVs to infer causality, further supports the hypothesis that higher HbA1c levels can lead to female infertility. The IVW method demonstrated a significant causal relationship, with an odds ratio of 1.82, indicating that genetically predicted higher HbA1c levels increase the risk of infertility. These findings align with prior research demonstrating the negative impact of diabetes and hyperglycemia on female fertility. For example, a study using the Skåne Healthcare Register, which examined 20 years of medical records, found that women diagnosed with type 2 diabetes before their reproductive years had significantly lower birth rates (62.6% vs. 83.8%), higher risks of miscarriage (RR = 1.88, 95% CI: 1.50–2.36), and increased infertility risks (RR = 3.44, 95% CI: 2.88–4.10) compared to women without diabetes, even after adjusting for factors such as PCOS and obesity [7]. Another Swedish study followed 5978 women hospitalized for type 1 diabetes before age 16 and found a standardized fertility ratio of 0.80 (95% CI: 0.77–0.82). Women with complications such as retinopathy, nephropathy, neuropathy, or cardiovascular issues had significantly lower fertility rates and higher rates of congenital malformations compared to the general population [8]. Further supporting the role of glycemic control in fertility, a meta-analysis of ten randomized controlled trials demonstrated that improving insulin sensitivity significantly increased clinical pregnancy rates in infertile women with PCOS, regardless of intervention type, with superior benefits observed in those without severe obesity [9]. These results suggest that managing insulin resistance is critical for improving fertility outcomes, aligning with evidence that hyperglycemia and insulin resistance adversely affect ovarian function.

Both type 1 diabetes mellitus (T1DM) and type 2 diabetes mellitus (T2DM) are strongly associated with various reproductive dysfunctions in women, including delayed onset of menarche, irregular menstrual cycles, hormonal imbalances, diminished ovarian reserve, sexual dysfunction, PCOS, and an increased likelihood of early menopause [3,10,11,12,13]. These factors collectively negatively impact fertility. The underlying pathophysiological mechanisms may involve the hypothalamic–pituitary–ovarian axis and metabolic factors. A review study indicated that insulin resistance is closely linked to female infertility, affecting oocyte development, embryo quality, hormone secretion, and implantation, while also increasing the risk of spontaneous abortion and adverse pregnancy outcomes [14]. Moreover, animal studies have provided mechanistic insights that further support these findings. Comparative studies using two different mouse models of T1DM (genetic AKITA and streptozotocin-induced) demonstrated impaired folliculogenesis, oogenesis, and preimplantation embryogenesis, with significantly reduced pre-implantation embryo quality in both models. The streptozotocin model, in particular, showed additional reproductive dysfunctions, such as reduced ovary size, decreased luteinizing hormone receptor expression, fewer corpora lutea, impaired oocyte maturation, and lower serum progesterone levels [15]. Similarly, a mouse model of T2DM revealed that impaired glucose metabolism led to decreased endometrial glycogen levels, which hindered embryo implantation. Insulin treatment partially resolved these implantation defects, whereas metformin improved blood glucose levels but did not significantly affect local endometrial glucose metabolism [16].

This study has several strengths, primarily the integration of observational data from the NHANES 2017–2020 with MR analysis. By comprehensively evaluating multiple factors and utilizing a large sample size, we were able to adjust for various confounders in multivariable regression models, providing robust statistical power to analyze the relationship between HbA1c levels and female infertility. Furthermore, the MR method helped mitigate unmeasured confounding factors and reverse causation bias, enhancing the reliability of our findings. However, there are several limitations to our study. First, the diagnosis of infertility relied on self-reported data from the NHANES Reproductive Health Questionnaire, which may lead to classification bias due to recall bias and inaccuracies in self-reported data. Second, while HbA1c is a reliable indicator of long-term glycemic control, it may not capture short-term fluctuations in blood glucose levels that could affect reproductive health. Additionally, a single measurement of HbA1c may not fully represent an individual’s long-term glycemic control. Our study included only women who participated in the NHANES and had complete data for HbA1c, hypertension, hyperlipidemia, and BMI, which might introduce selection bias and affect the generalizability of our findings. Despite adjusting for several known confounders, there is still the possibility of residual and unmeasured confounding factors (such as dietary habits and physical activity) influencing the relationship between HbA1c levels and infertility. Moreover, the study population primarily consisted of individuals from the United States, and the genetic analysis was limited to those of European descent, limiting the generalizability of the results to other ethnicities or populations. Future studies in more diverse populations are needed to validate our findings.

## 4. Materials and Methods

### 4.1. Cross-Sectional Study

#### 4.1.1. Study Population

This study utilized data from the National Health and Nutrition Examination Survey (NHANES) collected between 2017 and 2020. The NHANES is a continuous program operated by the National Center for Health Statistics (NCHS) at the Centers for Disease Control and Prevention (CDC), designed to assess the health and nutritional status of adults and children in the United States. It employs complex, multistage probability sampling designed to produce a representative sample of the civilian, non-institutionalized U.S. population. The survey uniquely combines interviews, which cover demographic, socioeconomic, dietary, and health-related questions, with physical examinations, including medical, dental, and physiological measurements, as well as laboratory tests conducted by highly trained medical personnel. Due to the COVID-19 pandemic, the NHANES program suspended field operations in March 2020. As a result, data collection for the 2019–2020 cycle was not completed. Therefore, data collected from 2019 to March 2020 were combined with data from the 2017–2018 cycle to form a nationally representative sample of NHANES data from 2017 to March 2020, pre-pandemic.

**Figure 4:**
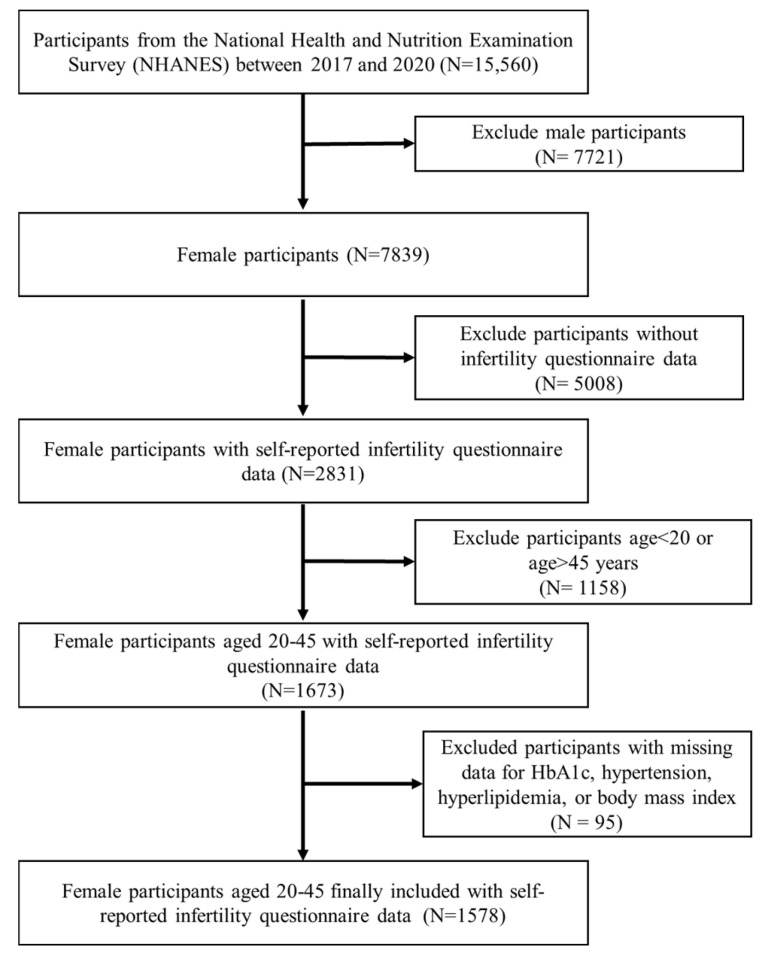
Flowchart of participant selection.

Women who participated in the NHANES during the March 2017–2020 cycle provided the data for this cross-sectional research. A total of 15,560 research participants were screened. Women aged 20–45 who answered “Have you ever attempted to become pregnant over a period of at least a year without becoming pregnant?” (*n* = 1673) were included in this study. We excluded participants with missing data for HbA1c (*n* = 80), hypertension (*n* = 2), hyperlipidemia (*n* = 4), and body mass index (BMI) (*n* = 9) at the time of the survey. In the end, 1578 participants were included in our research (Figure 4). All participants in the NHANES study signed informed consent forms authorized by the NCHS Research Ethics Review Board. For more detailed information about the NHANES survey, the public can access https://www.cdc.gov/nchs/nhanes/, accessed on 31 August 2024.

#### 4.1.2. Exposure and Outcome Definition

In this study, serum HbA1c levels were identified as the primary exposure variable. The HbA1c data were obtained from the NHANES 2017-March 2020 Pre-Pandemic Laboratory Data file named “Glycohemoglobin” (P_GHB). The detailed protocols for HbA1c measurements are documented on the official NHANES website.

The outcome variable, female infertility status, was determined based on self-reported data from the NHANES Reproductive Health Questionnaire (P_RHQ). Participants were asked the following question: “Have you ever attempted to become pregnant over a period of at least a year without becoming pregnant?” Women who answered “yes” to this question were classified as infertile, while those who answered “no” were classified as fertile.

#### 4.1.3. Assessment of Covariates

Covariates that might influence the relationship between HbA1c levels and infertility were also included in our analysis. Continuous variables consisted of age (years) and BMI (calculated by dividing an individual’s weight in kilograms by the square of their height in meters). Categorical variables included race/ethnicity (Mexican American, other Hispanic, non-Hispanic White, non-Hispanic Black, and other races), educational level (less than 9th grade, 9th–11th grade, high school or GED, some college or AA degree, and college graduate or above), marital status (married/living with partner, divorced, never married), hypertension, and hyperlipidemia.

#### 4.1.4. Statistical Analysis

Considering the complex survey design of the NHANES, we applied appropriate weights to each analysis to ensure our results accurately reflected nationally representative estimates. Categorical variables were presented as weighted proportions, while continuous variables were expressed as weighted means ± standard deviations (SD). Baseline characteristics between different groups were compared using the Chi-square test, Student’s t-test, or Fisher’s exact test, as appropriate.

We investigated the independent association between HbA1c levels and infertility using multivariable logistic regression in three distinct models:

Model 1: No covariates were adjusted.

Model 2: Adjusted for age, race/ethnicity, education level, and marital status.

Model 3: Adjusted for age, race/ethnicity, education level, marital status, BMI, hypertension, and hyperlipidemia.

Additionally, subgroup analysis was conducted based on diabetes diagnosis categorized as normal (<5.7%), prediabetes (5.7–6.4%), and diabetes (≥6.5%). All statistical analyses were performed using EmpowerStats (http://www.empowerstats.com, accessed on 31 August 2024), with the threshold for statistical significance set at *p* < 0.05.

### 4.2. Mendelian Randomization Study

#### 4.2.1. Study Design

**Figure 5:**
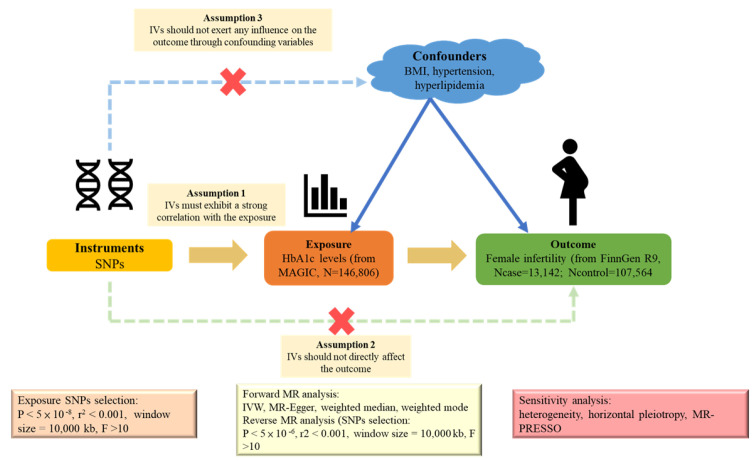
Schematic diagram of the two-sample Mendelian randomization framework used to assess the causal relationship between HbA1c levels and female infertility.

We designed our study as a two-sample bidirectional MR analysis, leveraging the strengths of the causal inference method. Using the publicly available summary statistics from two distinct genome-wide association studies (GWASs), we investigated the potential causal relationship between HbA1c and female infertility. We employed single-nucleotide polymorphisms (SNPs) as IVs. For MR validity, IVs must satisfy three key assumptions: (1) a strong correlation with exposure, (2) no influence on the outcome via confounding variables, and (3) no direct effect on the outcome [17]. A visual representation of the two-sample MR analysis is shown in Figure 5. As our data stemmed from publicly available online databases and the original studies obtained ethical approval and informed consent, our study did not require additional ethical approval or consent.

#### 4.2.2. Genome-Wide Association Study (GWAS) Sources

For our study, we utilized the most comprehensive and extensive genome-wide association study (GWAS) provided by the Meta-Analyses of Glucose and Insulin-Related traits Consortium (MAGIC), which encompasses data from 146,806 individuals of European descent, to examine exposures related to HbA1c [18]. MAGIC is a collective endeavor aimed at pooling data from various GWAS to uncover novel loci implicated in the regulation of blood sugar and metabolic characteristics.

GWAS summary statistics for female infertility were sourced from the latest release of the FinnGen consortium R9 data (accessible at https://storage.googleapis.com/finngen-public-data-r9/summary_stats/finngen_R9_N14_FEMALEINFERT.gz, accessed on 31 August 2024) [19]. This GWAS analysis involved 120,706 adult females from Finland, inclusive of 13,142 female infertility cases and 107,564 controls.

All SNPs and related data were obtained from studies that exclusively examined European populations, thus mitigating potential demographic stratification biases.

#### 4.2.3. Selection of IVs

In pursuit of suitable IVs, we used SNPs according to a rigorous criterion. Firstly, we considered only those SNPs that had reached the genome-wide significance level (*p* < 5 × 10^−8^). Subsequently, we conducted a linkage disequilibrium (LD) analysis, demanding an r^2^ value > 0.001 and a distance within 10,000 kb to guarantee the independence of our selected SNPs. Subsequently, we calculated the F-statistic for each IV to evaluate its strength and excluded weak instruments. We employed the following meticulous mathematical formula: F = R^2^ × (*n* − K − 1)/[K × (1 − R^2^)], where R^2^ represents the variance in exposure explained by each IV, *n* is the sample size of the GWAS, and K is the number of SNPs used in the MR analysis. Notably, all the IVs showed robust F-statistics, with values exceeding 10. Additionally, to harmonize our candidate variants with the outcome data and ensure robustness, we systematically removed palindromic SNPs consisting of one specific SNP (SNP:rs340882) without utilizing any proxy SNPs. In our pursuit of reliability, the filtered SNPs were subjected to the MR Steiger test to validate the causal direction of the IVs, and no SNPs were pruned during this step. Prior research has identified the key confounders, including the BMI, hypertension, and hyperlipidemia, in the association between HbA1c and female infertility [20,21,22,23]. Therefore, we meticulously examined the PhenoScanner V2 website (http://www.phenoscanner.medschl.cam.ac.uk/, accessed on 31 August 2024) and systematically excluded SNPs that were significantly associated (*p*  <  5 × 10^−5^) with these confounding variables. This led to the removal of four SNPs (rs11558471, rs7903146, rs1800562, and rs9376090). Finally, we employed MR pleiotropy residual sum and outlier (MR-PRESSO) methods to identify any outlier SNPs that might contribute to pleiotropy [24] and uncovered no outliers. For the reverse-direction MR analysis concerning the female infertility phenotype, the significance level was set similarly at 5 × 10^−6^, rather than the more commonly used 5 × 10 ^−8^, to compensate for the limited number of available instruments and ensure robust analysis despite this constraint [25], following analogous analytical steps.

#### 4.2.4. Bidirectional MR Analyses

We utilized an array of MR methods, including inverse variance-weighted (IVW), MR-Egger, weighted median, and weighted modes, to delve deeply into the potential causal link between HbA1c levels and female infertility. The IVW method capitalizes on its capacity to provide unbiased causality estimates based on the inverse of the outcome variance, assuming that the selected genetic variants are valid and free of pleiotropy [24]. Notably, IVW, which is well known for its superior statistical power, was adopted as the primary analysis method in this study. In instances where heterogeneity, defined as variability in the causal estimates derived from different genetic variants (instrumental variables), was detected among the instrumental variables, we employed the random-effect inverse-variance weighted (IVW-RE) method as the primary analysis. The IVW-RE model accounts for potential differences in effect sizes among the instrumental variables by incorporating a random-effect framework that allows for heterogeneity across these genetic instruments. Conversely, in cases where no heterogeneity was detected, we utilized the fixed-effect inverse-variance weighted (IVW-FE) method. The IVW-FE model assumes that all genetic variants have the same effect on the exposure and outcome, providing a more precise estimate when the effects are consistent and no heterogeneity is present [24]. The MR-Egger method was integrated with its distinct ability to detect and correct directional pleiotropy in MR analyses, offering unbiased causal estimates even when some IVs were invalid [26]. The weighted median method was employed, notable for its resilience, as it bases its causal estimates on the median instrument ratio and remains consistent, even if a significant fraction of the weight arises from invalid variants [27]. Finally, we applied the weighted mode method, focusing on the mode of the distribution of causal estimates from each genetic variant, ensuring reliability even when confronted with a majority of invalid genetic variants, as long as valid instruments predominantly determine the exposure variance [28].

#### 4.2.5. Sensitivity Analyses

Sensitivity analyses were conducted to determine the robustness of the MR results. The heterogeneity test (Cochran’s Q test in the IVW and MR-Egger method) was used to examine the differences between each IV; *p* < 0.05 was taken to represent the presence of heterogeneity [28,29]. In addition, MR-Egger regression was performed, which can be used to detect the directional pleiotropy of genetic variants. An intercept of zero for the regression equation or a non-significant *p*-value (*p* > 0.05) for the intercept term indicated that there was no average pleiotropic bias [28,30]. Finally, a leave-one-out analysis was performed to evaluate whether the MR results were strongly driven by a specific SNP [31].

#### 4.2.6. Statistical Analyses

All statistical analyses were performed based on the “MendelianRandomization” (version 0.9.0), “TwoSampleMR” (version 0.5.6) and “MRPRESSO” (version 1.0) packages in RStudio (R version 4.3.0, R Project for Statistical Computing).

## 5. Conclusions

In conclusion, our study provides evidence that elevated HbA1c levels are associated with an increased risk of female infertility, highlighting the importance of glycemic control in reproductive health management. Future research should focus on longitudinal studies to confirm these findings and explore the underlying mechanisms. Additionally, investigating the impact of glycemic control interventions on infertility outcomes could provide valuable insights for clinical practice. Overall, this study emphasizes the need for healthcare providers to consider glycemic control as a crucial factor in managing reproductive health in women. Monitoring and managing HbA1c levels may not only improve overall health but also enhance reproductive outcomes.

## Figures and Tables

**Table 1:** Baseline characteristics of the study population according to female infertility.

Characteristics	Female Infertility		*p*-Value
	No (*n* = 1386)	Yes (*n* = 192)	
**Age (year)**	32.22 ± 7.65	33.46 ± 6.35	0.036
**Race/Hispanic origin, (%)**			0.358
Mexican American	11.78	11.27	0.710
Other Hispanic	8.43	11.16	
Non-Hispanic White	56.14	56.97	
Non-Hispanic Black	13.1	11.97	
Other race—including multi-racial	10.55	8.63	
**Education level, (%)**			0.489
Less than 9th grade	2.72	1.77	
9–11th grade	6.36	9.44	
High school graduate/GED or equivalent	21.49	22.78	
Some college or AA degree	32.49	32.4	
College graduate or above	36.94	33.61	
**Marital status, (%)**			<0.001
Married/living with partner	56.83	79.14	
Widowed/divorced/separated	8.44	7.36	
Never married	34.73	13.5	
**BMI (kg/m^2^)**	29.36 ± 8.48	32.76 ± 9.04	<0.001
**Hypertension, (%)**			0.589
Yes	11.79	10.43	
No	88.21	89.57	
**Hyperlipidemia, (%)**			0.015
Yes	13.46	20.19	
No	86.54	79.81	
**HbA1c (%)**	5.32 ± 0.59	5.57 ± 0.99	<0.001

Mean ± SD for continuous variables: the *p* value was calculated by the weighted linear regression model. (%) for categorical variables: the *p* value was calculated by the weighted chi-square test. BMI = body mass index, HbA1c = glycated hemoglobin.

**Table 2:** The associations between HbA1c and infertility.

Exposure	Model 1 ^1^ [OR (95% CI)], *p*-Value	Model 2 ^2^ [OR (95% CI)], *p*-Value	Model 3 ^3^ [OR (95% CI)], *p*-Value
HbA1c (%) (continuous)	1.48 (1.21, 1.81), <0.001	1.57 (1.25, 1.98), <0.001	1.40 (1.15, 1.69), 0.003
HbA1c (%) (categories)			
<5.7	Reference	Reference	Reference
5.7–6.4	1.73 (1.09, 2.75), 0.029	1.78 (1.03, 3.07), 0.040	1.34 (0.76, 2.36), 0.261
≥6.5	3.62 (1.82, 7.20), 0.001	4.43 (2.03, 9.67), 0.001	3.32 (1.50, 7.36), 0.007

In the sensitivity analysis, HbA1C was converted from a continuous variable to a categorical variable. OR, odds ratio; 95% Cl, 95% confidence interval. ^1^ Model 1: no covariates were adjusted. ^2^ Model 2: adjusted for age, race, education level, and marital status. ^3^ Model 3: adjusted for age, race, education level, marital status, body mass index, hypertension, and hyperlipidemia. HbA1c = glycated hemoglobin.

**Table 3:** Heterogeneity and directional pleiotropy test results in Mendelian randomization analysis.

Heterogeneity Test					
Exposure	Outcome	Method	Q	Q_df	Q_pval
HbA1c	Female infertility	MR-Egger	12.21	15	0.66
		Inverse-variance weighted	13.69	16	0.62
Female infertility	HbA1c	MR-Egger	26.25	21	0.20
		Inverse-variance weighted	26.31	22	0.24
**Directional pleiotropy test**					
**Exposure**	**Outcome**	**Egger intercept**	**SE**	***p*-value**	**Global test’s *p*-value of MR-PRESSO**
HbA1c	Female infertility	0.012	0.01	0.24	0.66
Female infertility	HbA1c	−0.0002	0.001	0.83	0.22

HbA1c = glycated hemoglobin, Q = Cochran’s Q statistic, Q_df = degrees of freedom for Q, SE = standard error, MR-PRESSO = Mendelian Randomization Pleiotropy Residual Sum and Outlier.

## Data Availability

The data in this study are available to other researchers upon request.

## Supplementary Materials

The following supporting information can be downloaded at: https://www.mdpi.com/article/10.3390/ijms25179668/s1.
